# Challenges and facilitators to parent–child shared physical interventions during preschool and school age education: a systematic review and meta synthesis

**DOI:** 10.3389/fpubh.2025.1658179

**Published:** 2025-11-05

**Authors:** Lijuan Xiong

**Affiliations:** College of Education and Psychological Sciences, Sichuan University of Science & Engineering, Zigong, China

**Keywords:** challenges, facilitators, physical activity, children, parents, education

## Abstract

**Background:**

Despite strong evidence supporting physical activity’s role in children’s physical, cognitive, and emotional development, many preschool and school-aged children fail to meet recommended activity levels. Parent–child shared physical activity interventions offer a promising approach, yet their implementation is influenced by multiple contextual factors. This study aimed to explore the challenges and facilitators of parent–child shared physical activity interventions in preschool and school-aged education settings.

**Methods:**

A comprehensive literature search was conducted across six databases—PubMed, Scopus, Web of Science, PsycINFO, Embase, and Google Scholar—using a combination of MeSH terms and keywords related to children, parents, shared physical activity, and implementation factors. Inclusion criteria focused on qualitative or mixed-method studies involving parents, educators, or stakeholders of children aged 3–11 years engaged in shared physical activity. Studies were screened and appraised using the CASP checklist. Qualitative data were analyzed using conventional content analysis, allowing for the identification of themes and subthemes.

**Results:**

Four high-quality studies met the inclusion criteria. Two overarching categories emerged: challenges and facilitators. Key challenges included environmental and Structural Barriers, parental attitudes and low engagement, child-related challenges to participation and sociocultural and cognitive Influences on participation. Facilitators encompassed motivational strategies to encourage participation, program design that supports family engagement, strengthened parent–child relationships and institutional and environmental enablers.

**Conclusion:**

Parent–child shared physical activity interventions are most effective when they are culturally appropriate, developmentally suitable, and adaptable to daily routines. Overcoming structural and behavioral barriers through coordinated support from families, educators, and communities is essential for their long-term success.

## Introduction

Physical activity is crucial for pre-school and school-aged children, not only for physical health but also for cognitive development, particularly executive functioning (1, 2). Regular physical activity is positively linked to improved executive functions, such as working memory, inhibitory control, and cognitive flexibility. These skills are essential for academic success, problem-solving, and self-regulation (3). The Canadian Physical Activity Guidelines for Children and Youth, along with numerous similar recommendations globally, such as those from Australia, the USA (as outlined by the US Department of Health and Human Services in 2008), and the World Health Organization (WHO in 2010), advise that children and young people should engage in at least 60 min of moderate- to vigorous-intensity physical activity (MVPA) daily (4).

Despite these recommendations, data consistently reveals that a substantial proportion of children across various age groups fail to meet these guidelines (3, 5–8).

Different countries have adopted various strategies to promote physical activity among children. For instance, Capelle et al.’s systematic review in Australia identified several effective approaches for improving preschoolers’ fundamental motor skills, including movement-based (like running, jumping, throwing, catching, kicking, and balancing), parent-involved, combined, and play-based interventions (9). Parents exert a lasting influence on their preschool children’s behavior development, potentially shaping it throughout their entire lives. Consequently, to effectively encourage physical activity in preschool children and adolescents, researchers are increasingly focusing on understanding parents’ physical activity habits and levels (10). While schools offer a valuable setting for promoting activity (8), the profound and enduring influence of parents on their children’s behavioral development, potentially lasting a lifetime, necessitates a deeper understanding of the familial context of physical activity (11, 12). Considering the impact of parent-related behaviors—such as parental support, attitudes towards physical activity, and role modeling—on children’s physical activity, findings indicate that young children’s physical activity behavior is particularly and significantly shaped by their parents’ beliefs, attitudes, and actions (12). Parents’ levels of MVPA have a notable connection with their preschool children’s total physical activity (TPA) levels; however, this link tends to become less strong as the child ages (11). A study in Japan by Tanaka et al. (2018) revealed that children whose mothers attended their sports events engaged in significantly more MVPA compared to children whose mothers did not. Interestingly, paternal support showed no association with children’s MVPA levels (13).

Given the established critical role of physical activity in the holistic development of preschool and school-aged children, encompassing not only their physical health but also their cognitive capacities, particularly executive functions vital for academic and life success, the persistent challenge of low physical activity levels is a significant concern (3). The existing research highlights the potential of parent-focused interventions to improve children’s motor skills and physical activity. However, the success of these interventions depends on addressing challenges and utilizing facilitators within the parent–child relationship. Understanding barriers like time constraints and facilitators like enhanced personality development and leadership skills in children is crucial for designing effective strategies (13). The existing research highlights the potential of parent-focused interventions to improve children’s motor skills and physical activity. However, the success of these interventions depends on addressing challenges and utilizing facilitators within the parent–child relationship. Understanding barriers like time constraints and facilitators like enhanced personality development and leadership skills in children is crucial for designing effective strategies (10). Although previous studies (14) have examined parental involvement in children’s physical activity, these contributions are often context-specific or descriptive in scope. This review advances the field by integrating evidence across multiple cultural and educational contexts and synthesizing findings through an inductive meta-synthesis. By organizing barriers and facilitators into a socioecological framework and pairing them with practical facilitators, this study provides both theoretical innovation and actionable guidance for intervention design and policy development. This article aimed to determine challenges and facilitators of parent–child shared physical activity during pre-school and school-aged education.

## Methods

### Study design

A meta-synthesis method was used to integrate and interpret findings from multiple qualitative studies, providing a comprehensive understanding of the phenomenon. The review followed the Preferred Reporting Items for Systematic Reviews and Meta-Analyses (PRISMA) guidelines, which ensure transparent and standardized reporting (15).

### Inclusion criteria

Studies were eligible for inclusion in this review if they met the following criteria:

Eligible participants included parents, caregivers, educators, and other stakeholders engaged in joint physical activities with children aged 3–11 years. Interventions were broadly defined as any structured or unstructured movement activity undertaken by parents (or caregivers) and children together, either in the home, community, early childhood education centers, or school settings. Only qualitative or mixed-methods studies with a clearly identifiable qualitative component, such as interviews, focus groups, or thematic/content analysis, were considered. To be included, studies had to provide evidence on perceived or observed factors influencing the implementation, participation, or effectiveness of shared parent–child physical activity. Publications were restricted to peer-reviewed articles in English. No limitations were imposed on the date of publication to ensure comprehensive coverage. Mixed-methods studies were only included if their qualitative elements directly addressed the research question and met the eligibility requirements.

### Exclusion criteria

Research that addressed only individual physical activity of either the parent or the child, without an element of joint participation, was not eligible. Similarly, studies involving infants, adolescents, or populations outside the defined preschool and school-age range were excluded. Studies that reported solely on outcomes or effectiveness without examining implementation-related experiences were not considered. Purely quantitative studies, theoretical papers, opinion pieces, conference abstracts, and literature reviews were also excluded. In addition, studies conducted exclusively in clinical or medical settings such as hospitals or rehabilitation centers were omitted unless they were directly relevant to educational or home-based contexts. To ensure methodological rigor, only peer-reviewed publications were included, while dissertations, theses, book chapters, and other forms of grey literature were excluded.

### Search strategy and screening

A comprehensive literature search was conducted across six electronic databases—PubMed, Scopus, Web of Science, PsycINFO, Embase, and Google Scholar—covering all records from the beginning to June 2025. The initial strategy combined keywords and MeSH terms across four main concepts: children (e.g., “child,” “preschooler,” “toddler,” “young child”), parents (e.g., “parent,” “guardian,” “mother,” “father,” “family”), shared physical activity (e.g., “shared physical intervention,” “shared activity,” “dyadic movement,” “co-active movement”), and facilitators/challenges (e.g., “enablers,” “barriers,” “obstacles,” “opportunities,” “focus group,” “phenomenology”). To improve specificity and exclude non-research articles, we applied filters to remove systematic reviews, meta-analyses, and narrative reviews. In the final stage, the search was refined to focus primarily on terms related to shared physical activity while maintaining the exclusion of review articles, ensuring that only primary studies directly relevant to the research question were retrieved.

**Figure 1:**
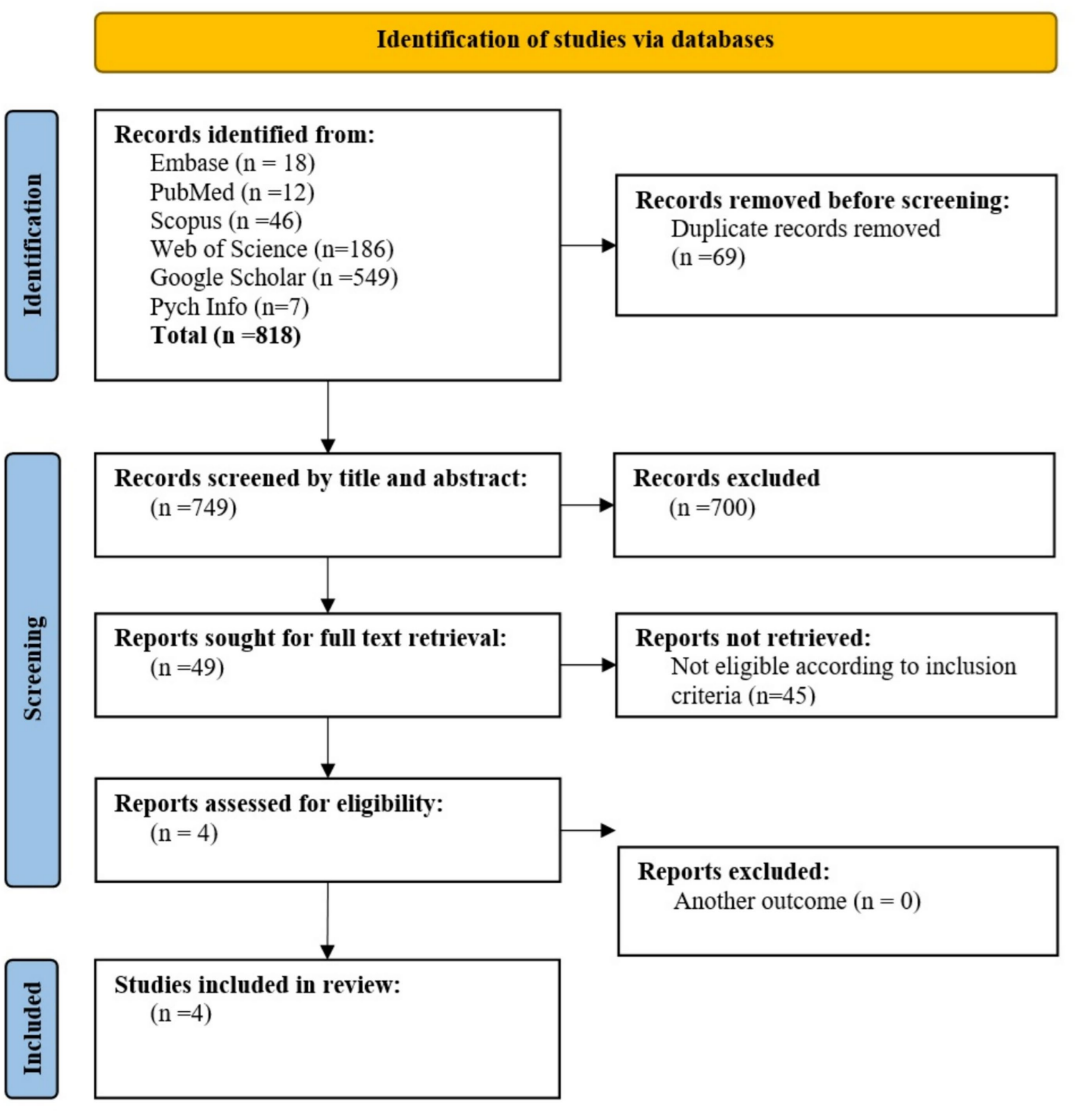
Flowchart of screening process.

The screening of studies was conducted solely by the author, guided by the inclusion and exclusion criteria described above. In the first phase, titles and abstracts were reviewed to assess eligibility, after which potentially relevant studies were retrieved for full-text screening. Each article was carefully evaluated against the predefined criteria, and decisions regarding inclusion or exclusion were made systematically and consistently. A total of 818 studies proceeded to full-text review, after which 16 met the eligibility requirements and were included in the final meta-synthesis. A summary of the screening process, along with reasons for exclusion at the full-text stage, is presented in Figure 1 (PRISMA flow diagram). Key characteristics of the included studies are provided in Table 1. This review was not registered in PROSPERO, as it focused on the synthesis of qualitative evidence rather than a quantitative systematic review or meta-analysis.

**Table 1:** Information extracted from the articles reviewed in this meta-synthesis.

Author(s) (Year) Country	Title	Type of study	Participants/number	Setting and format for data collection	The data analysis	Results	Quality (CASP Score)
Kippe (2025) (18) Norway	The importance of preschool employees’ individual and shared opinions for 4–6-year-olds’ physical activity in preschool – in light of individual and collective identity	Qualitative study	Preschool teachers and assistants/*n* = 13	Focus group interviews	Coding qualitative data	Diverse individual and collective perspectives on children’s physical activity were observed across preschools. In two of the three settings, a stronger alignment between personal and shared views highlighted the value of fostering a unified culture to minimize conflicts among staff and support consistent promotion of physical activity.	High (8.5)
Larsen et al. (2004) (20) The United States	Barriers to Physical Activity Qualitative Data on Caregiver–Daughter Perceptions and Practices	Qualitative study	Girls and their primary female caregivers/*n* = 23	Face-to-face semi structured, in-depth interviews	Coding qualitative data, including double coding	Barriers to physical activity: perceived lack of affordable, accessible recreation facilities and low caregiver motivation. Potential intervention strategies: walking for exercise, transportation and several low-cost, favored physical activities, such as hopscotch, jumping rope, and dance.	High (8)
Rhodes et al. (2017) (21) Canada	Promoting Parent and Child Physical Activity Together: Elicitation of Potential Intervention Targets and Preferences	Online survey	Parents with Children/*n* = 483	Not mentioned	Coding qualitative data	Behavioral beliefs about health, interpersonal, educational/learning opportunities, control beliefs about lack of time, various incompatible parent/child factors, parental health and bad weather	High (9)
Al-walah et al. (2024) (10) Saudi Arabia	Barriers, enablers and motivators of the “I’m an active Hero” physical activity intervention for preschool children: a qualitative study	Qualitative study	Preschool principals, preschool staff members and parents/*n* = 15	Semi-structured interviews	Thematic analysis	(1) Barriers to parental involvement in preschool PA interventions, such as time constraints, lack of flexibility, limited space, and a shortage of trained staff; (2) Risks and benefits of children’s programmed participation; (3) Motivators including rewards, nonfinancial incentives, and concerns about childhood obesity and a sedentary lifestyle; (4) Facilitating factors for overcoming barriers, including staff training, time reallocation, staff coordination, space optimization, non-financial incentives, and sustaining partnerships.	High (8.5)

### Data extraction

A data extraction table was developed specifically for this study, drawing on formats successfully used in previous meta-syntheses. Data was extracted on study characteristics, participant profiles, intervention features, and the reported challenges and facilitators, together with related outcomes (see Table 1). Qualitative data were extracted from the results or findings sections of each included study. All extracted data were imported into MAXQDA 20 software to support systematic coding and thematic synthesis.

### Quality assessment

The first reviewer performed an initial search for relevant articles across various databases, including PubMed, Scopus, Web of Science, Psych Info, Embase, and Google Scholar and imported them into EndNote (version) to manage citations and remove duplicates. Two independent authors screened titles and abstracts against the inclusion criteria. Subsequently, full-text reviews were conducted for the remaining articles, and those not meeting the criteria were excluded. Any disagreements arising during the article selection process were settled through discussion with the second reviewer. The Critical Appraisal Skills Programme (CASP), a 10-item quality assessment tool for qualitative research, was used to evaluate study quality. Studies were classified as high quality (8 + criteria met), medium quality (5–7 criteria met), or low quality (4 or fewer criteria met) (16).

### Data synthesis

The data for this systematic review and meta-synthesis were analyzed using conventional qualitative content analysis, as outlined by Lindgren et al. (17). This interpretive approach was chosen to ensure a rigorous and transparent process of identifying and synthesizing patterns within qualitative findings, rather than employing numerical modeling or simulation-based methods.

All extracted findings were read repeatedly to gain a deep understanding of the contextual factors influencing parent–child shared physical interventions. Segments of text directly related to barriers or enablers were identified as meaning units. These units were then condensed while preserving their core meaning and assigned initial codes. Code sharing conceptual similarities were grouped into broader categories through an iterative process of comparison and refinement.

Two overarching categories (Challenges and Facilitators) emerged, each encompassing several descriptive subthemes that reflected the lived experiences and perspectives reported across the included studies. This inductive synthesis provided an integrated understanding of the factors influencing the implementation and effectiveness of parent–child shared physical interventions in preschool and school-age settings.

## Results

### Presentation of studies

This meta-synthesis included four high-quality studies conducted in diverse cultural and educational contexts including Norway, the United States, Canada, and Saudi Arabia. The studies employed qualitative or mixed method designs and used varied data collection methods, including focus group interviews, semi-structured interviews, and online surveys. Participants included parents, preschool staff, and primary caregivers of children aged 3 to 11 years. Sample sizes were between 13 and 483 participants (Table 1).

### Description of the themes

Two Categories emerged from the analysis: (1) Challenges (Environmental and Structural Barriers, Parental Attitudes and Low Engagement, Child-Related Challenges to Participation, Sociocultural and Cognitive Influences on Participation) and (2) Facilitators (Motivational Strategies to Encourage Participation, Program Design That Supports Family Engagement, Strengthened Parent–Child Relationships, Institutional and Environmental Enablers). Table 2 presents a detailed overview of the identified themes and subthemes. The following are the two categories and their themes and sub-themes:

**Table 2:** Overview of categories, themes and sub-themes.

Categories	Themes	Sub-themes	Quotes
Challenges	Environmental and structural barriers	- Space and safety constraints - Time constraints and daily life priorities - Resource limitations	1. “*the preschool children play in the sports hall once in a while, but there is no point in being there for a long time. All toys and equipment are gone after 3 p.m. Then they run more, more ‘catch and run’. Furthermore, indoor running was not allowed in the preschool. It is a common rule for everyone*.” (18) 2. *“(The traffic) is very bad*... *to be honest that’s why I do not walk a lot,” “I do not feel safe... people fly up and down the hill and act like the children are not there,” and “We have also posted on our street ‘no 18-wheeler trucks,’ but they continue to come up the street.*” (19) 3. “*So it’s [TV] pretty much to pacify them [the children] while I’m getting something done*.” (20) 4. “*Lack of time (56%) and occupational work (20%) were the most common subthemes*.” (21) 5. *“I have tried to get [my daughter] in several activities at the park recreation department. They do not get enough kids so she cannot get involved because enough kids do not sign up.”* Another stated, *“[I would like to see] a lot more organization and a lot more availability for minorities. There is nothing around here at all. And what there is to do, half the time we cannot afford it.”* (20) 6. “*J Just as the program is engagingly simplified, it should also be financially viable. We should try to replicate the simplicity in the games to make it affordable in terms of material cost, which should not be above the reasonable financial capacity of the preschool.*” (10)
	Parental attitudes and low engagement	- Inactive lifestyles and lack of motivation - Personal beliefs that undermine coactivity	7. “*I’m the one who is lazy, and I cannot make her run outside and make her do exercise while I sit there and watch TV. So, I cannot blame her if she wants to come inside and watch TV because she learns from example*.” (20) 8. 23% of parents endorsed fatigue as a barrier, saying they were “*too tired, exhausted*.” Others noted, “*I enjoy being myself and sitting still*.” (21) 9. “*If they sit and enjoy themselves in role play or interaction in play, I think it is more important than that they should run*.” (18) 10. “*I say honestly, I tell you that I am surprised now that sport is important for children at this early age. I mean, I expected that sports are for older children, but the small and skinny ones also need sports! [I thought that] as long as he plays at home, then his things are fine*.” (10)
	Child-related challenges to participation	- Preference for sedentary entertainment - Lack of shared interests or enjoyment - Reluctance due to physical unease and fear of harm	11. “*Oh yeah, she’d find something else to do. If I said ‘no TV,’ they would run to that computer*.” (20) 12. “*Parents’ perception of incompatible aspects between themselves and their children was endorsed by over a third of parents (34%).” This included “activity differences (11%), low interest in participating in activities (8%), or a general unwillingness to be active with the child (11%)*.” (21) 13. “*As a mother, I have observed that children in kindergarten are at risk…they are prone to falling unless they possess a good understanding (perception). If engaging in sports is necessary, both the teacher and the mother should be present*.” (10)
	Sociocultural and cognitive influences on participation	- Lack of developmental understanding of physical activity - Cultural norms that discourage active family engagement - Insufficient training and capacity among educators	14. “*I say honestly, I tell you that I am surprised now that sport is important for children at this early age. I mean, I expected that sports are for older children, but the small and skinny ones also need sports!*” (10) 15. “*We have a social life that is sacred over any other commitment. I mean, like father and mother, they are busy with whirlpools throughout the week, and after this, [the] weekend is a life of social engagement*.” (10) 16. “*I expect that what can hinder PA [include], for example, you have time parameters. Are they committed to a predetermined academic timetable? Is there also a limitation in classroom space, or …? The number of students has also increased*.” (10)
Facilitators	Motivational strategies to encourage participation	- Positive reinforcement through incentive-based strategies - Motivation through structured play and collaborative challenges	17. “*Children love to be rewarded, even with simple things, and to have rewards from the things they love. I mean, for example, some children love to hear stories … we saturate them with books from which they can benefit*.” Another parent explained, “*At home with my children, I award a star to the child who consistently brushes their teeth. This practice fosters regularity and commitment. Similarly, we implemented this approach in ‘I am the hero’ program*.” (10) 18. “*Instil in the child the spirit of leadership; for instance, they might be told, ‘Today, you will lead us in a game. Choose a game, gather us together, and facilitate the game*.” Teachers also emphasized that “*Conducting competitions among the children in kindergarten and at home*” was a motivating factor that increased active participation. (10)
	Program design that supports family engagement	- Child-friendly and culturally relevant activities - Flexibility in scheduling and format - Ease of implementation in home and community settings	19. Children expressed enthusiasm for familiar activities, saying they liked “*hopscotch, jumping rope, dancing, swimming, and bicycling*.” (20) 20. “*The ideas of these games should be well-suited to the space of the house and the age of the preschool child*.” (10) 21. “*Most parents preferred to keep coactivity outside, within the backyard (81%) or local park (75%), and with all family members included (77%).*” (21) 22. “*We can adjust the class… set a simplified corner in the classroom… or allocate it in the kindergarten courtyard*.” (10) 23. “*We might walk to the store once or twice a week together*,” (21)
	Strengthened parent–child relationships	- Emotional bonding through shared movement - Modeling of active lifestyles	24. “*From my point of view, the involvement of parents in this program with their children is very important, as it will strengthen the child’s personality… and strengthen the child’s bond with their parents*.” (10) 25. “*Children by their nature like to imitate their parents*.” (10)
	Institutional and environmental enablers	- Active involvement of educators - Safe and stimulating physical environments - Accessible guidance and educational materials	26. “*I emphasize its importance and I see it as an easy program. It will be easy to apply… one of the most important factors is that it is inexpensive*.” (10) 27. “*We have selected what we like to do, cow barns and outdoor activities… we spend most of the day outdoors*.” (18) 28. “*Most wanted the activity… promoted by fitness trained professionals (71%) or the family physician (39%) via Internet (54–57%)*.” (21)

### Challenges of parent–child shared physical interventions

#### Environmental and structural barriers

##### Space and safety constraints

Limited space in classrooms and homes, unsafe outdoor environments, and weather conditions posed significant physical barriers to activity. These constraints hindered children’s ability to play freely and reduced opportunities for shared movement in safe, comfortable settings. Caregivers in articles reported that their neighborhoods were considerably non conducive to an active lifestyle (see quotes 1–2 in Table 2) (18, 19).

##### Time constraints and daily life priorities

Parents frequently cited being overburdened with work, school responsibilities, or family obligations, leaving little time for coactivity. Despite understanding its importance, physical activity was often deprioritized in daily routines. Caregivers reported that TV filled an important role as a “baby-sitter,” indicating no advantages for turning the TV off (10, 20, 21) (see quotes 3–4 in Table 2).

##### Resource limitations

Financial and infrastructure limitations affected access to organized programs and facilities. The caregivers were particularly concerned about the financial cost, viewing it as a potential risk factor for the discontinuation of the program. Some families could not afford formal physical activities or lived in areas where recreational resources were lacking or inaccessible (10, 20) (see quotes 5–6 in Table 2).

#### Parental attitudes and low engagement

##### Inactive lifestyles and lack of motivation

Parental fatigue, disinterest, or a generally sedentary lifestyle were commonly cited as barriers to physical engagement. Parents who were not active themselves struggled to motivate their children, acknowledging that their own habits influenced their child’s behavior (20, 21) (see quotes 7–8 in Table 2).

##### Personal beliefs that undermine coactivity

Some parents undervalued shared physical activity, believing unstructured play or rest was more appropriate. Others did not recognize the importance of PA in early childhood or believed their children were too young or healthy to need formal exercise (10, 18) (see quotes 9–10 in Table 2).

#### Child-related challenges to participation

##### Preference for sedentary entertainment

Children often preferred sedentary activities, particularly screen-based entertainment such as watching television or playing video games. This preference made it difficult for parents to motivate their children to engage in physical activity, even when encouragement was provided. In one study, most girls indicated that they did not like to play outdoors and preferred to watch TV. Caregivers also stated that although they try to encourage their girls to play outside, they cannot get them to go outside (20) (see quote 11 in Table 2).

##### Lack of shared interests or enjoyment

Differences in preferences and interests between parents and children often created a disconnect that reduced shared participation in physical activity. Some parents noted that their children preferred being active with peers or found joint activities with parents less engaging (21) (see quote 12 in Table 2).

##### Reluctance due to physical unease and fear of harm

Fear of physical harm, such as falling during activity, discouraged both parents and children from engaging fully in physical tasks. Parents, particularly mothers, expressed concerns over safety which often limited active participation or required high supervision (10) (see quote 13 in Table 2).

#### Sociocultural and cognitive influences on participation

##### Lack of developmental understanding of physical activity

Some parents lacked awareness of how physical activity contributes to developmental domains such as motor skills, cognitive growth, and emotional well-being. This lack of understanding sometimes led to underestimating the importance of shared physical play, especially at a young age (10) (see quote 14 in Table 2).

##### Cultural norms that discourage active family engagement

In some cultural contexts, social norms favored passive or indoor family time over physical activity. Family obligations, traditions, and social life often took precedence over scheduled or structured physical movement (10) (see quote 15 in Table 2).

##### Insufficient training and capacity among educators

Educators faced barriers such as overcrowded classrooms, tight academic schedules, and lack of training, which limited their ability to support physical activity. The lack of institutional flexibility made it difficult to implement coactivity-focused interventions consistently (10) (see quote 16 in Table 2).

### Facilitators of parent–child shared physical interventions

#### Motivational strategies to encourage participation

##### Positive reinforcement through incentive-based strategies

Using tangible and intangible rewards like stickers, stories, or simple praise helped motivate children to engage in shared physical activities. These strategies made the experience enjoyable, reinforced effort, and fostered behavioral consistency over time. Parental insights highlighted how such methods could be tailored to suit children’s interests and developmental levels (10) (see quote 17 in Table 2).

##### Motivation through structured play and collaborative challenges

Physical activities that involved family-based games, challenges, or friendly competitions boosted enthusiasm and made exercise more appealing. Children felt empowered when they were allowed to take initiative or leadership roles during these activities, enhancing their engagement and sense of accomplishment (10) (see quote 18 in Table 2).

#### Program design that supports family engagement

##### Child-friendly and culturally relevant activities

Activities that were age-appropriate, culturally familiar, and matched children’s interests were more successful in engaging families. These forms of movement were already part of children’s play routines and required minimal instruction or equipment (10, 20) (see quotes 19–20 in Table 2).

##### Flexibility in scheduling and format

Parents preferred physical activity programs that were adaptable to their routines, typically in the afternoons or evenings. They also favored non-formal settings like backyards or parks, which made participation more feasible and enjoyable (21) (see quote 21 in Table 2).

##### Ease of implementation in home and community settings

Interventions that required little preparation, cost, or travel were especially appreciated by parents. Simple adjustments to the home or classroom environment allowed families and educators to support physical activity (10, 21) (see quotes 22–23 in Table 2).

#### Strengthened parent–child relationships

##### Emotional bonding through shared movement

Engaging in physical activity together provided a space for emotional connection, shared joy, and mutual understanding. These moments strengthened the parent–child bond and increased children’s sense of belonging and support (10) (see quote 24 in Table 2). In one study, 37% of parents agreed that coactivity provided quality family bonding time, creating closeness and stronger relationships (21).

##### Modeling of active lifestyles

When parents actively participated in physical activity, they modeled healthy behavior for their children. Children often mirrored their parents’ actions, which increased their motivation and normalized an active lifestyle (10) (see quote 25 in Table 2). In one study, 20% of parents stated that coactivity allowed them to model lifelong healthy behaviors and lead by example (21).

#### Institutional and environmental enablers

##### Active involvement of educators

Support from teachers and school leaders improved children’s enthusiasm for physical activity. Educators who supported or implemented the program enhanced its impact and sustainability (10) (see quote 26 in Table 2).

##### Safe and stimulating physical environments

Access to open, safe, and natural spaces such as parks and schoolyards encouraged regular physical activity. These environments stimulated curiosity and engagement (18) (see quote 27 in Table 2).

##### Accessible guidance and educational materials

Simple and clear instructions or materials—delivered online or through trusted individuals—helped families understand and participate in coactivity. Preferred sources included fitness professionals, family physicians, and internet-based platforms (21) (see quote 28 in Table 2).

## Discussion

This meta-synthesis elucidates the complex web of factors that influence parent–child shared physical activity interventions. By integrating findings from caregivers, educators, and children, a comprehensive understanding emerged of how socioecological, behavioral, and institutional dynamics jointly shape engagement. While coactivity demonstrates strong potential to enhance health, emotional bonding, and family cohesion, its implementation is constrained by structural, personal, and systemic barriers. Importantly, the findings highlight that the success of such interventions depends on how different methodological approaches—environmental, behavioral, and institutional—are applied and aligned with real-world contexts.

From an environmental perspective, the inadequacy of physical spaces represented one of the most consistent barriers to shared activity. Families often cited lack of access to safe, child-friendly spaces—especially in urban or underserved settings—as a deterrent to engagement. Concerns regarding traffic, neighborhood safety, and inadequate public facilities echoed previous evidence that the built environment, including walkability, recreational infrastructure, and perceived safety, plays a critical role in determining physical activity opportunities (19, 22, 23). Environmental conditions not only shape accessibility but also influence the quality and spontaneity of physical engagement. Less visible factors—such as heat, air pollution, and urban congestion—further restrict outdoor activity (24, 25). Therefore, interventions aimed solely at modifying the physical environment, though essential, remain insufficient if not complemented by behavioral and motivational components. In practice, strategies such as improving public play areas or classroom layouts must be integrated with family education and community-based programs to ensure consistent utilization and long-term adherence.

Time scarcity also emerged as a major barrier across studies. Caregivers juggling work, household duties, and multiple responsibilities struggled to incorporate physical activity into daily routines. This limitation reflects broader societal trends where sedentary coping mechanisms, such as screen use, replace active engagement. Consistent with earlier research (26), time-related barriers are often both practical and psychological: caregivers perceive physical activity as competing with other priorities rather than being an integral part of family life. Hence, behavioral interventions that embed short, manageable activities—such as active commuting or playful household tasks—are more likely to succeed than those demanding substantial schedule changes. Tailored strategies that normalize movement as a family routine may therefore hold greater practical feasibility than purely environmental reforms.

The family and behavioral dimension proved central to both challenges and facilitators. Parents’ own activity levels, beliefs, and motivational states profoundly influenced their children’s participation. Some caregivers underestimated the developmental importance of physical activity, viewing it as secondary to academic achievement or rest. This aligns with existing literature linking parental modeling and health beliefs with children’s activity behaviors (27–29). Moreover, high parental screen use and sedentary patterns—documented as strong correlates of children’s inactivity (30–34)—illustrate how interventions must address parental habits alongside those of children. Behavioral intervention frameworks that emphasize role modeling, positive reinforcement, and shared play represent a cost-effective and context-sensitive strategy, especially in settings where structural improvements are not immediately feasible.

Children’s increasing preference for sedentary entertainment, particularly screen-based activities, compounds this challenge. According to Behavioral Choice Theory, individuals naturally gravitate toward the most rewarding leisure option (35, 36). In contemporary households, digital activities often provide greater immediate gratification than physical play (37–39). Effective intervention design must therefore recalibrate the reinforcement structure by making physical activity more intrinsically rewarding. Gamification, family-based challenges, and cooperative play can help shift motivation from passive entertainment toward active participation—aligning enjoyment with health-promoting behavior.

Another recurring issue was safety concern. Caregivers frequently limited outdoor activity due to fears of injury, traffic, or stranger danger, which is consistent with research linking perceived neighborhood safety to child activity levels (40–42). These anxieties underline the importance of environmental safety interventions such as supervised play programs, well-maintained parks, and structured group activities. Yet, as this synthesis indicates, improving environmental safety alone is not enough; caregivers’ perceptions must also be addressed through education, reassurance, and trust-building mechanisms delivered via schools or community networks.

Cultural norms and cognitive perceptions also shaped family attitudes toward coactivity. In several households, passive or academic family time was prioritized over physical engagement, and some parents expressed limited awareness of the developmental benefits of physical activity. These findings align with prior work showing that health literacy and developmental knowledge influence family health behaviors (43–45). Consequently, educational interventions that raise awareness of the cognitive, emotional, and social benefits of movement may help reframe physical activity as an essential developmental practice rather than a discretionary leisure pursuit.

At the institutional level, structural and curricular barriers constrained implementation in educational settings. Overcrowded classrooms, limited space, rigid academic schedules, and lack of teacher training frequently restricted opportunities for movement integration. These findings mirror studies from Asian contexts that emphasize the interplay between school infrastructure, policy priorities, and physical fitness outcomes (46–48). Institutional change—through flexible scheduling, professional development, and policy mandates—thus constitutes a crucial enabling condition for sustaining behavioral interventions. Institutional-level approaches ensure that the benefits of family and environmental strategies are reinforced within formal educational systems, creating consistency across home and school contexts.

Despite these challenges, numerous facilitators were identified that can inform future intervention design. Motivation-enhancing techniques—such as positive reinforcement, structured play, and child-led challenges—proved effective in promoting engagement. Grounded in self-determination theory, these strategies cultivate autonomy, competence, and intrinsic motivation (49). Prior evidence confirms that when teachers or facilitators provide constructive feedback and enjoyable experiences, participation, effort, and affective outcomes improve significantly (50, 51). Similarly, gamification and small rewards can stimulate self-efficacy, which, in turn, reinforces sustained engagement in physical activity.

Programmatic characteristics also influenced success. Interventions that were culturally relevant, developmentally appropriate, and flexible in timing achieved higher adherence. Activities that resembled children’s natural play patterns—such as running, jumping, or tag games—required minimal equipment and were easier to sustain (52). These design principles align with applied performance models, suggesting that interventions embedded in everyday routines, rather than imposed as structured sessions, are more sustainable in family life.

Beyond physical outcomes, coactivity strengthened the emotional and relational bonds between parents and children. Shared activities provided avenues for connection, modeling, and mutual enjoyment, extending the benefits of physical activity into psychological well-being and family cohesion (53). These interpersonal gains serve as a motivational anchor that can sustain participation over time and across contexts.

Finally, institutional and community supports were pivotal in sustaining engagement. Educators and coaches who actively endorsed physical activity fostered enthusiasm and normalized active behaviors (54–56). Institutional involvement ensures continuity between home, school, and community, promoting a culture where movement is valued and practiced across environments. Therefore, a multilevel, integrated approach—combining environmental modifications, behavioral engagement, and institutional reinforcement—appears most effective for enhancing both participation and sustainability.

### Implications for policy and practice

The present findings underscore that selecting and applying the appropriate intervention method should depend on both the setting and the target population’s resources and capacities. Policymakers should prioritize interventions that integrate structural feasibility with behavioral engagement. For example, in communities with limited public spaces, policies that promote home-based coactivity and use of public parks can address environmental barriers. In contrast, school systems should emphasize teacher training and flexible scheduling to operationalize institutional support for daily movement.

Practically, interventions that rely on behavioral change (such as parental modeling, motivational reinforcement, and shared activity planning) offer higher potential for sustained engagement and should be prioritized when resources for structural reform are limited. Moreover, integrating these behavioral methods into existing institutional frameworks can amplify their impact. Urban planners, educators, and public health officials should therefore collaborate to align environmental modifications (safe spaces), behavioral programs (parental involvement), and institutional mechanisms (policy-based scheduling and training).

By adopting this integrated, context-sensitive approach, practitioners and policymakers can enhance the long-term success and scalability of parent–child shared physical activity interventions across diverse educational and socioeconomic settings.

### Limitations

This review has several limitations that should be acknowledged. First, only four studies met the inclusion criteria, which limits the breadth of evidence and the generalizability of the findings. The small sample of included studies, although high in quality, restricts the ability to draw conclusions across diverse educational systems, cultural contexts, and socioeconomic groups.

Second, the exclusive inclusion of English-language, peer-reviewed publications may have introduced language and publication bias, potentially omitting relevant studies published in other languages or from the grey literature. This may have excluded culturally specific perspectives from non-English-speaking regions, which are particularly important in family-based and culturally sensitive interventions.

Third, although mixed-methods studies were considered, only those with a substantial qualitative component were included, which may have limited the range of reported outcomes and perspectives. Additionally, variations in methodological approaches across studies—such as differences in data collection tools, analytic frameworks, and participant demographics—may have introduced inconsistencies in theme development and synthesis.

Finally, the reliance on self-reported perceptions and retrospective accounts from parents, educators, and caregivers may have been subject to recall or social desirability bias. This could affect the accuracy of reported challenges and facilitators, particularly in studies involving sensitive or socially influenced topics like parental engagement and home environments.

Future research should include more diverse and longitudinal studies to capture a broader range of experiences and evolving factors.

## Conclusion

The challenges and facilitators identified in this synthesis underscore that promoting parent–child shared physical activity requires a comprehensive, systems-level approach. Addressing environmental, behavioral, and institutional barriers while leveraging motivational strategies and supportive infrastructures can significantly enhance the feasibility and impact of coactivity interventions. Integrating these insights into public health initiatives and early education policy offers a promising pathway toward cultivating active, connected, and developmentally enriched family environments.

This review is distinctive in that it provides one of the first qualitative meta-syntheses focused explicitly on family coactivity, synthesizing evidence across multiple cultural and educational contexts. By applying an interpretive, socioecological framework, it extends previous research that emphasized isolated or quantitative findings, offering a deeper theoretical and methodological understanding of how families, educators, and institutions can jointly support sustainable physical activity engagement in early childhood.
